# Effective of different industrial disinfection in subzero cold-chain environment

**DOI:** 10.1038/s41598-024-62204-x

**Published:** 2024-06-02

**Authors:** Zhe Ren, Jie Han, Xue Zhang, Zheng Yan, QiuHua Wei

**Affiliations:** 1 The Chinese PLA Center for Disease Control and Prevention, Beijing, China; 2 Tianjin Medical University, Tianjin, China

**Keywords:** Cold chain, Disinfection, Pathogens, Microbiology, Chemical biology

## Abstract

Effective disinfection methods are crucial in the cold chain transportation process of food due to the specificity of temperature and the diversity of contaminated flora. The objective of this study was to investigate the sanitizing effect of different disinfectants on various fungi at – 20 °C to achieve accurate disinfection of diverse bacterial populations. Peracetic acid, hydrogen peroxide, and potassium bisulfate were selected as low-temperature disinfectants and were combined with antifreeze. The sanitizing effect of these cryogenic disinfectants on pathogens such as *Bacillus subtilis black variant spores* (ATCC9372), *Staphylococcus aureus* (ATCC 6538), *Candida albicans* (ATCC 10231), *Escherichia coli* (8099), and *poliovirus* (PV-1) was sequentially verified by bactericidal and virus inactivation experiments. After a specified time of disinfection, a neutralizing agent was used to halt the sanitizing process. The study demonstrates that different disinfectants exhibit selective effects during the low-temperature disinfection process. Peracetic acid, hydrogen peroxide, and potassium monopersulfate are suitable for the low-temperature environmental disinfection of bacterial propagules, viruses, and fungal contaminants. However, for microorganisms with strong resistance to spores, a low-temperature disinfectant based on peracetic acid should be chosen for effective disinfection treatment. Our results provide a valuable reference for selecting appropriate disinfectants to sanitize various potential pathogens in the future.

## Introduction

The food cold chain is a refrigerated supply chain used for maintaining perishable products at low temperatures during production, storage, and distribution, with the objective of reducing losses while preserving the quality and freshness of the food and drug products^1^. The cold chain is critical for ensuring food safety due to the ability of freezing temperatures to inhibit the growth of bacteria and viruses; most viral pathogens linked to the food cold chain impact the gastrointestinal system and are commonly associated with undercooked food, rather than respiratory illnesses^2,3^. The objective of our study is to highlight that conventional disinfection measures may be inadequate or absent in critical aspects of the frozen food cold chain environments and operations. Recent studies have shown that human respiratory viruses can be transmitted through frozen food, emphasizing the need for more effective disinfection measures to establish a safer cold chain^4^.

The most direct and effective means of eradicating the virus is through the use of disinfectants. Disinfectants are highly efficacious in impeding the transmission of the virus by sanitizing surfaces, objects, and the environment. The effectiveness of common chemical disinfectants decreases significantly with decreasing temperature. In fact, some disinfectants that are highly effective at normal temperatures become ineffective or unsuitable when subjected to freezing conditions. Consequently, the development of new low-temperature disinfectants has become a pressing need to combat the spread of the new coronavirus. Cryogenic disinfection refers to the sterilization of an object or environment using temperatures below 0 °C. Low-temperature disinfectants are formulated by adding antifreeze agents to conventional chemical disinfectants, and are capable of effectively disinfecting surfaces and objects under low-temperature conditions^5^. In response to the coronavirus situation, China government has issued a number of technical guidelines on disinfecting the cold chain used in food production^6^. These guidelines specify that disinfection should be conducted at critical steps and efficacy should be verified, but they do not provide explicit instructions on how to perform low-temperature disinfection. Therefore, it can be said that disinfection measures in the frozen food chain are still being explored and optimized. There is currently no evidence to suggest that traditional disinfection methods are effective at low temperatures, and data is required to validate their efficacy.

Peracetic acid (PAA) and hydrogen peroxide (H_2_O_2_) belong to the group of peroxide disinfectants^7,8^. Since PAA has strong oxidizing and weak acidic material characteristics, it has a strong damaging effect on bacterial propagules and spores. Therefore, PAA is used as a high-efficiency disinfectant, even as a sterilization agent^9^. Meanwhile, H_2_O_2_ is widely used as an environmental-friendly and efficient disinfectant because its decomposition products are non-toxic and harmless^10^. Another new type of relatively stable and less corrosive disinfectant, potassium peroxymonosulfate (PMPS), is widely used in the disinfection of drinking water due to its small by-products, and has gradually attracted attention in the sterilisation industry^11–13^.

Microbial resistance to chemical biocides and sterilization processes varies widely^14^. Resistance to sterilization in microorganisms primarily stems from surface protection mechanisms, which are based on differences in the composition of the outer shells among different types of microorganisms. The hierarchy of microbial resistance to sterilization typically follows the order of spores, fungi, Gram-negative bacteria, Gram-positive bacteria, and enveloped viruses^15^. Consequently, the subsequent study necessitates an investigation into the bactericidal efficiency of different disinfectants under low-temperature conditions, utilizing microorganisms representing various levels of sensitivity as representatives. Our study systematically studies the three disinfectants (PAA, H_2_O_2_, and PMPS) against gram-positive bacteria, negative bacteria, fungi, viruses, and spores at low temperatures. Our data can support a wise choice of usage of specific disinfectants in a cold chain environment.

## Materials and methods

### Bacterial strains, virus strains, and cell lines

The strains used for testing included *Bacillus subtilis black variant spores* (ATCC 9372), *Staphylococcus aureus* (ATCC 6538), *Candida albicans* (ATCC 10231), and vero cells (ATCC CCL-81), all of which were procured from the American Type Culture Collection (ATCC) in the United States. *Poliovirus* (PV-1) and *Escherichia coli* (8099) were provided by the Disinfection and Infection Control Department of the PLA Center for Disease Control and Prevention in China. All experimental procedures were conducted in a secondary biosafety laboratory.

### Bacterial proliferation and preparation of flakes

*Staphylococcus aureus* and *Escherichia coli* were cultured at 37 °C for 18–24 h using TSA beveled culture, and then rinsed with PBS and diluted to the required concentration. The diluted bacteria were mixed 1:1 with 3% BSA, aspirated in 20 µL suspension droplets on a 1 cm × 1 cm plastic film, and used after drying for approximately 15 min at room temperature. The amount of bacteria used in the neutralizing agent carrier test was sufficient to ensure that the amount of recovered bacteria fell between 2.5 × 10^2^ CFU/tablet and 1.5 × 10^3^ CFU/tablet. The number of recovered bacteria per tablet in the vector immersion quantitative sterilization test ranged from 1 × 10^6^ CFU/tablet to 5 × 10^6^ CFU/tablet^8^. *Candida albicans* was cultured using Sar’s inclined medium in the same manner as the preparation of flakes. *Bacillus subtilis black variant spores* were inoculated in Roche bottles containing TSA, cultured in a constant temperature incubator at 37 °C for 5–7 days, and then stained for spores. When the spore formation rate exceeded 90%, spores were recovered and stored in a 4 °C refrigerator for future use. Bacillus tablets were prepared as for *E. coli* tablets.

### Virus propagation and preparation of *Poliovirus* strain

The frozen *poliovirus* strain was thawed in a water bath at 37 °C, suitably diluted with cell maintenance solution, and inoculated into a flask previously covered with a monolayer of Vero cells. The flask was then placed in an incubator with 5% carbon dioxide and cultured at 37 °C until cytopathic effects (CPE) were observed daily. Once CPE was observed in 75% of cells, the cells were freeze-thawed three times (alternating between − 20 and 37 °C), and the virus suspension was aspirated into a centrifuge tube. The suspension was then centrifuged at 6000 r/min for 10 min to remove the precipitate. The virus infection titer TCID50/0.1 mL was determined following the 2002 edition of “Disinfection Technical Specifications”, and the titer was found to be greater than 10^7^, which was deemed suitable for testing. The PV-1 strain was diluted 1:1 in pairs with 3% BSA, and 20 µL of suspension droplets were stained on a 1 cm × 1 cm plastic film, dried, and set aside for further experiments.

### Composition of low-temperature disinfectants

The antifreeze was composed of a 2:1 volume ratio of ethanol to propylene glycol. The disinfectant and antifreeze were combined in a 2:1 ratio, resulting in a final concentration of 3% H_2_O_2_, 0.1% PAA, and 6% PMPS. Once mixed, the disinfectant was stored in a refrigerator at – 18 °C for at least 8 h to prevent crystallization before usage.

### Bactericidal experiments

Each sterilized plastic film was completely immersed in 5 mL of low-temperature disinfectant, and the action was applied for a specified time. The plastic film was then removed with sterile forceps and placed into a test tube containing 5 mL of neutralizing agent, mixed, and diluted by a factor of 10 if necessary. Then, 1 mL of the extracted liquid was transferred into a sterile dish, and tryptic soy agar (TSA) medium was poured and the microbial culture media was allowed to solidify. The dish was incubated at 37 °C, and the results were observed after 48 h. The trial was repeated three times.

### Virus inactivation experiments

The virus-infected plastic film carrier was completely immersed in a low-temperature disinfectant, and the action was applied for a specified time. The plastic film was then removed with sterile forceps and placed into a test tube containing 2 mL of cell maintenance solution, mixed throughly, and 0.1 mL of suspension was pipetted into a sterile EP tube containing 0.9 mL of cell maintenance solution. The suspension was then diluted in a tenfold series, and each dilution was seeded in four 96-well plates filled with monolayers of cells. The plates were then cultured in a carbon dioxide incubator at 37 °C with 5% CO_2_, and CPE (PV-I vaccine strain for 3 d) was observed daily at the same time to calculate the logarithmic value of viral TCID50. The trial was repeated three times.

### Data analysis

The disinfection efficacy of PAA, H_2_O_2_, and PMPS for bacterial pathogens was assessed by calculating the Log10 colony forming units (CFU). Reduction of ≥ 3 Log10 CFU/mL was considered to be sufficient disinfection. Figures were plotted with GraphPad software (8.0). The difference between each group were calculated with an one-way ANOVA analysis. The significance of the results was shown as *, **, *** indicating *P* < 0.05, 0.01, and 0.001, respectively.

## Results

### Effectiveness of three disinfectants on bacterial disinfection at low temperatures

**Figure 1:**
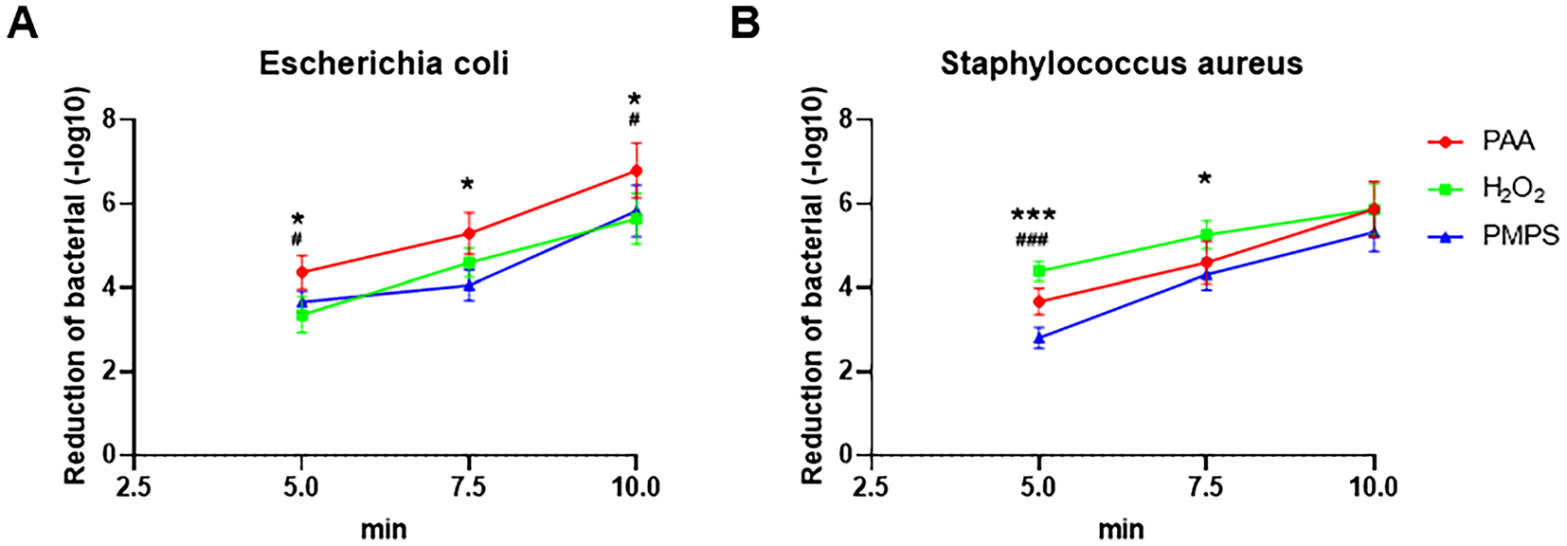
The disinfected effectivity of PAA, H_2_O_2_, and PMPS on *Escherichia coli* (**A**) and *Staphylococcus aureus* (**B**) under low-temperature conditions. * Indicates the comparison of PAA and PMPS, and # indicates the comparisons of PAA and H_2_O_2_. * or ^#^ *P* < 0.05.

We conducted an analysis of the efficacy of various disinfectants on *Staphylococcus aureus* and *Escherichia coli* under low-temperature conditions (Fig. 1). Our findings indicate that all three disinfectants (PAA, H_2_O_2_, and PMPS) exhibited strong bactericidal properties, as evidenced by logarithmic values exceeding 3.0 after just 2.5 min of antibacterial treatment. Furthermore, a notable increase in the efficacy of the three disinfectants was observed after 7.5 min of treatment. Specifically, PAA exhibited the highest killing efficiency against *E. coli* at 5 min, with a corresponding efficiency of 6.79 at 10 min. Morever, the highest killing efficiency for H_2_O_2_ against *S. aureus* was observed at 5 min (4.24 efficiency), with an absolute value of 5.87 at 10 min.

### Effectiveness of three disinfectants on PV-1 disinfection at low temperatures

**Figure 2:**
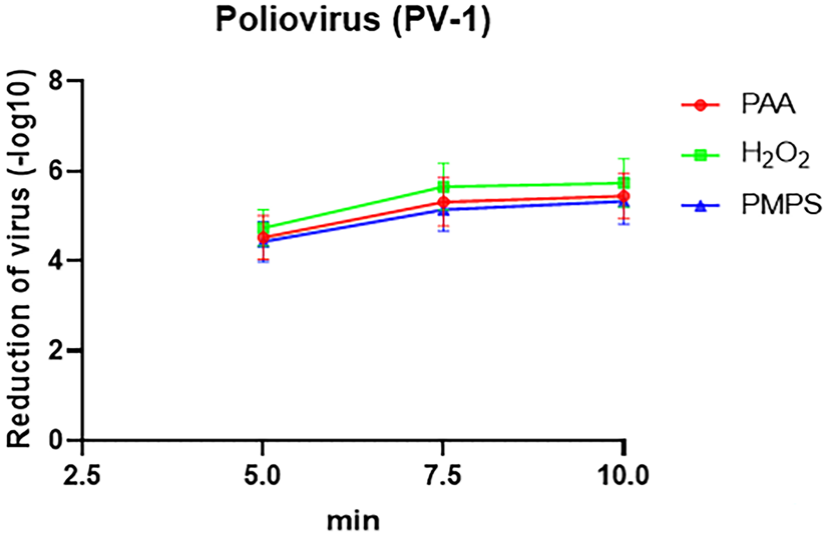
The effectivity of PAA, H_2_O_2_, and PMPS on PV-1 under low-temperature conditions.

We analyzed the disinfection effect of different disinfectants on PV-1 under low-temperature conditions (Fig. 2). Our findings revealed that all three disinfectants had a significant disinfecting effect on PV-1 after a 5-min disinfecting treatment calculated by Log10 CFU/mL (PAA = 4.52, H_2_O_2_ = 4.73, and PMS = 4.42). Furthermore, the highest level of disinfecting efficiency was achieved when the three disinfectants were applied for a duration of 10 min. Notably, all three disinfectants exhibited equal efficacy in killing the virus under low-temperature conditions.

### Effectiveness of three disinfectants on fungus disinfection at low temperatures

**Figure 3:**
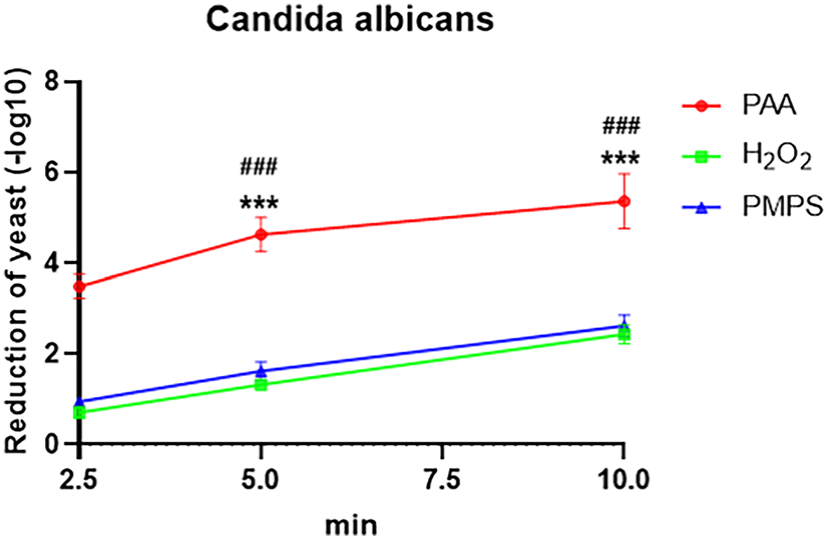
The effectivity of PAA, H_2_O_2_, and PMPS on *Candida albicans* under low-temperature conditions. * Indicate the comparison of PAA and PMPS, and ^#^ indicates the comparisons of PAA and H_2_O_2_. *** or ^###^ *P* < 0.001.

We also analyzed the disinfection effect of the three different disinfectants on *Candida albicans* under low-temperature conditions (Fig. 3). The results showed that PAA was highly effective against *Candida albicans*; after 5 min of disinfection, all logarithmic values were greater than 3.0. After 10 min of treatment, the absolute value of disinfection was 5.36. Meanwhile, H_2_O_2_ and PMPS were less effective against *Candida albicans*, as the absolute values of disinfection of H_2_O_2_ and PMPS were 2.44 and 2.62, respectively.

### Effectiveness of three disinfectants on spores disinfection at low temperatures

**Figure 4:**
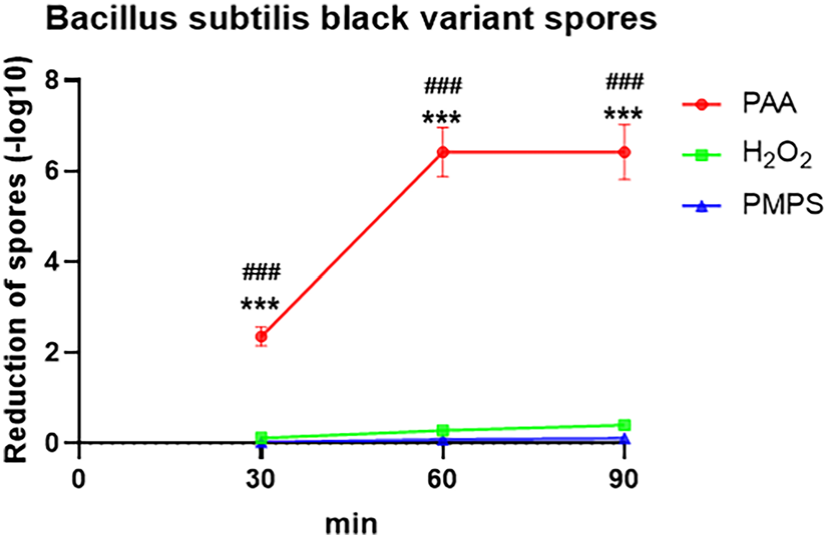
The effectivity of PAA, H_2_O_2_, and PMPS on *Bacillus subtilis black variant spores* under lowtemperature conditions. * indicate the comparison of PAA and PMPS, and ^#^ indicates the comparisons of PAA and H_2_O_2_. *** or ^###^ *P* < 0.001.

Spores are the most tolerant cells that have been found in nature. Spores in a dormant state are extremely resistant to sterilization such as heat, drying, radiation, acids, bases, and organic solvents^16^. We analyzed the disinfection effect of three disinfectants on *Bacillus subtilis black variant spores* under low-temperature conditions (Fig. 4). The results showed that PAA had a significantly positive effect on killing *Bacillus subtilis black variant spores*: after 30 min of disinfection, the absolute value of disinfection was 2.2. After 90 min of PAA treatment, the absolute value of disinfection reached a maximum of 6.11. Contrastingly, H_2_O_2_ and PMPS had no disinfection effect on *Bacillus subtilis black variant spores*.

## Discussion

Oxidizing sterilants primarily destroy pathogenic components by disrupting DNA or RNA structures, inhibiting protein enzyme activities, and damaging lipids through the generation of oxygen radicals^17^. The antimicrobial efficacy of oxidizing agents is significantly influenced by temperature; elevated temperatures enhance the rate of chemical reactions involving the sterilant, thereby accelerating the interactions between the sterilant and microbial cellular components. Currently, there is a lack of systematic studies on the efficacy of various types of disinfectants against different pathogens at low temperatures^18,19^.

In the context of regulatory requirements, both the Environmental Protection Agency (EPA) of the United States and the Biocidal Products Regulation (BPR) of the European Union acknowledge the importance of broad-spectrum activity in disinfectants. When manufacturers seek registration for specific types of disinfectants, such as bactericides, fungicides, virucides, or sporicides, regulatory agencies often require testing against only a limited number of representative species from each category^20^. This study described here adopts a comprehensive approach, encompassing a range of microbial pathogens including *Bacillus subtilis black variant spores, Staphylococcus aureus, Candida albicans, Escherichia coli, and poliovirus*. This selection is informed by various factors, including the relevance of these microbes to real-world scenarios such as healthcare, food production, and exposure in public spaces, as well as the need to assess the disinfectant's efficacy against diverse types of microorganisms^21^. For example, *Staphylococcus aureus* is a common bacteria found on the surface of food and can cause infections, so this bacteria is often included in studies on the evaluation of surface disinfectants. *E. coli* is a common bacterium found in the intestines of humans and animals and can be highly transmissible through fecal contamination, making it important in studies related to sanitation. *Bacillus subtilis* spores are commonly used as a surrogate for more resistant spore-forming pathogens, while *Candida albicans* represents fungal pathogens, and poliovirus represents viruses.

PAA disinfectants have been used in the food industry and by water or wastewater treatment companies, as well as in the decontamination and sterilization of heat-sensitive medical and hospital equipment and installations^22,23^. The PAA substance acts quickly and is effective against bacteria, fungi, viruses, and spores. PAA is effective over a wide temperature range (0–40 °C) and pH (3.0–7.5)^24^. However, whether PAA also has a sanitizing role in low-temperature environments has not been systematically studied. Studies have proved that PAA can effectively inactivate *Porcine Epidemic Diarrhea virus* (PEDV), *Simian immunodeficiency virus* (SIV), *E. coli*, *Staphylococcus aureus*, and *Bacillus subtilis endo sanitize* spores at a low-temperature of – 20 °C^25–27^. Our research proves that PAA has a statistically significant killing effect on various pathogenic microorganisms such as gram-positive bacteria, negative bacteria, fungi, viruses, and spores under low-temperature conditions.

Similar to PAA, H_2_O_2_ is also a peroxide disinfectant. H_2_O_2_ can sanitize a variety of pathogens such as viruses, bacteria, fungi, and spores. Due to its benign decomposition into non-toxic byproducts, H_2_O_2_ finds extensive application in sterilizing environments, including food processing and medical device sanitation. However, there is currently no research to prove that H_2_O_2_ can effectively sanitize PEDV virus on aluminum surfaces in a freezing environment^28^. The sanitizing efficiency and selection of H_2_O_2_ for fungi and spores’ pathogens at low temperatures have not been systematically studied. Here, our research demonstrated that H_2_O_2_ in combination with antifreeze could efficiently disinfect viruses, bacteria, fungi, and spores pathogens at low-temperature.

Another disinfectant we studied, PMPS, is the potassium salt of peroxymonosulfuric acid, which is widely used as an oxidizing agent. This salt probably acts on bacteria by oxidation. It also attacks viral protein capsids, thereby releasing and inactivating the nucleic acids of viruses, thus affecting the bactericidal and virucidal efficacies under various concentrations, exposure or contact times, and organic material conditions^11^. PMPS shows efficient disinfection ability against bacteria and viruses on a variety of materials^11,13,29^. Here, our results demonstrated that PMPS could efficiently disinfect viruses, bacteria, and fungi pathogens at low-temperature. However, PMPS could not disinfect spores under low-temperature conditions.

In conclusion, this study underscores the critical importance of effective disinfection methods within the cold chain transportation process of food, where precise temperature control and diverse microbial contamination pose significant challenges. Our investigation into the sanitizing effect of three different disinfectants at – 20 °C reveals promising results. PAA, H_2_O_2_, and PMPS, when combined with antifreeze, demonstrate selective efficacy against a range of pathogens including bacterial propagules, viruses, and fungal contaminants. Importantly, our findings suggest that peracetic acid-based disinfectants are particularly effective against microorganisms with strong resistance to spores. These insights offer valuable guidance for selecting appropriate disinfection strategies to combat diverse microbial threats in low-temperature environments, providing a foundation for enhanced food safety and public health measures within the cold chain.

This study represents the inaugural systematic evaluation of the efficacy of sterilizing agents under low-temperature conditions against a spectrum of microorganisms ranging from spores to viruses. Its comprehensive scope encompasses various types of target microorganisms, providing a foundational framework for the subsequent selection of sterilizing agents tailored to low-temperature settings. However, the research methodology predominantly focuses on the analysis of single-agent concentrations. Future investigations should encompass orthogonal analyses involving varying concentrations and types of cryoprotectants to ascertain optimal sterilizing conditions for each disinfectant under low-temperature conditions and their respective application scenarios.

## Data Availability

Data supporting the findings of this study are available from the corresponding author upon reasonable request.
